# Stable Cytotoxic T Cell Escape Mutation in Hepatitis C Virus Is Linked to Maintenance of Viral Fitness

**DOI:** 10.1371/journal.ppat.1000143

**Published:** 2008-09-05

**Authors:** Luke Uebelhoer, Jin-Hwan Han, Benoit Callendret, Guaniri Mateu, Naglaa H. Shoukry, Holly L. Hanson, Charles M. Rice, Christopher M. Walker, Arash Grakoui

**Affiliations:** 1 Department of Medicine, Division of Infectious Diseases, Microbiology and Immunology, Emory Vaccine Center, Emory University School of Medicine, Atlanta, Georgia, United States of America; 2 Laboratory of Virology and Infectious Disease, Center for the Study of Hepatitis C, The Rockefeller University, New York, New York, United States of America; 3 The Center for Vaccines and Immunity, Nationwide Children's Hospital, The Ohio State University, Columbus, Ohio, United States of America; 4 Deparment of Pediatrics, The Ohio State University, Columbus Ohio, United States of America; 5 Department of Medicine, University of Montreal and Centre de Recherche du Centre Hospitalier de l'Université de Montréal (CR-CHUM), Montréal, Québec, Canada; University of Lausanne, Germany

## Abstract

Mechanisms by which hepatitis C virus (HCV) evades cellular immunity to establish persistence in chronically infected individuals are not clear. Mutations in human leukocyte antigen (HLA) class I-restricted epitopes targeted by CD8^+^ T cells are associated with persistence, but the extent to which these mutations affect viral fitness is not fully understood. Previous work showed that the HCV quasispecies in a persistently infected chimpanzee accumulated multiple mutations in numerous class I epitopes over a period of 7 years. During the acute phase of infection, one representative epitope in the C-terminal region of the NS3/4A helicase, NS3_1629-1637_, displayed multiple serial amino acid substitutions in major histocompatibility complex (MHC) anchor and T cell receptor (TCR) contact residues. Only one of these amino acid substitutions at position 9 (P9) of the epitope was stable in the quasispecies. We therefore assessed the effect of each mutation observed during in vivo infection on viral fitness and T cell responses using an HCV subgenomic replicon system and a recently developed in vitro infectious virus cell culture model. Mutation of a position 7 (P7) TCR-contact residue, I1635T, expectedly ablated the T cell response without affecting viral RNA replication or virion production. In contrast, two mutations at the P9 MHC-anchor residue abrogated antigen-specific T cell responses, but additionally decreased viral RNA replication and virion production. The first escape mutation, L1637P, detected in vivo only transiently at 3 mo after infection, decreased viral production, and reverted to the parental sequence in vitro. The second P9 variant, L1637S, which was stable in vivo through 7 years of follow-up, evaded the antigen-specific T cell response and did not revert in vitro despite being less optimal in virion production compared to the parental virus. These studies suggest that HCV escape mutants emerging early in infection are not necessarily stable, but are eventually replaced with variants that achieve a balance between immune evasion and fitness for replication.

## Author Summary

Hepatitis C virus (HCV)-associated liver disease is a leading indication for liver transplantation in the United States. With more than 170 million people infected with HCV worldwide and more than 70% of those infected unable to clear the virus, it is of paramount importance to elucidate the factors leading to viral persistence. A well-characterized experimental chimpanzee chronically infected with HCV was found to develop multiple viral sequence variations over the course of 7 years. Serial mutations in an HCV epitope that was originally immunogenic in the host were observed during the course of infection, and we sought to better understand how each mutation affected viral persistence. We recreated the viral variants detected in the chimpanzee and assessed the ability of each variant to replicate, produce progeny virus, and evade the host immune system. We found that certain HCV variants present long after initial infection are able to avoid recognition by host immune cells, but have reduced replication fitness. Importantly, in the absence of immune pressure, these viruses mutate back to variants better able to replicate and produce new viral progeny. Our experiments suggest that rapidly mutating viruses like HCV seek a balance between replication efficiency and the ability to evade the host immune system.

## Introduction

Hepatitis C virus (HCV) currently infects an estimated 3% of the world's population (∼170 million people) [1],[2], causing a myriad of health problems including fibrosis and cirrhosis of the liver [3],[4]. Infection considerably increases the probability of hepatocellular carcinoma, and HCV-related hepatic disease has become the leading cause of orthotopic liver transplantation in the United States [5]. The majority of those infected with the virus are unable to spontaneously resolve the infection despite the presence of humoral and cellular immune responses that are at least occasionally robust [6]–[8]. There have been many reasons proposed as to why the immune system fails in the face of chronic HCV infection, including early T cell exhaustion, particularly of the CD4+ helper subset [9],[10], dendritic cell (DC) dysfunction [11],[12], impairment of effector cells [6],[13],[14], and cytotoxic T lymphocyte (CTL) viral epitope escape [15]–[18]. Like most small RNA viruses, HCV has an extremely high replication rate (∼10^10^–10^12^ virions/d, [19]), and the highly error prone NS5B polymerase allows for robust production of minor viral variants that may outpace cellular immune responses [6],[20],[21]. These variants are under constant immune pressure in the infected host, and Darwinian selection processes lead to domination of the viral quasispecies by the most fit virus that can also evade immune recognition.

Viremia varies widely in individuals with chronic HCV infection with steady state values that range from a few thousand to several million genomes per milliliter of plasma. Factors that regulate virus load in persistent HCV infection are not known but could conceivably influence the rate and severity of progressive liver disease. CTL mutational escape could have positive or negative effects on virus replication depending on the site and nature of the amino acid substitution(s) within structural or non-structural HCV proteins. Some substitutions might be expected to result in loss of immune control and thus higher levels of virus production, but it is also plausible that mutations facilitating CTL escape have negative consequences for replication if they impair production, assembly, or release of virions. Impaired replicative fitness as a result of escape mutation has been associated with reduced viremia and slower disease progression in HIV-1-infected humans and SIV-infected rhesus macaques [22]–[25]. Despite the importance of CTL epitope viral mutation for immune evasion, in HCV infection many highly targeted epitopes have a low mutation frequency. Epitopes such as HLA-A2 restricted NS3_1073–1081_ are consistently targeted by CD8+ T cells, but amino acid mutations facilitating immune evasion are rarely observed [26],[27]. Since the NS3 protein shares both protease and NTPase-dependent helicase functions, it has been proposed that mutations in these epitopes may be lethal to the virus [28]. However, few studies have examined how CTL escape directly correlates with HCV fitness. Cell culture models of HCV replication utilizing viral replicons have been valuable in identifying adaptive mutations that facilitate robust replication in hepatocytes in vitro [29],[30]. Using this tool along with recently developed systems allowing actual infection rather than just replication [31]–[34], we extend these models to study the impact of CTL escape mutation on virus replication and virion production. In this report, we assessed the evolution of a dominant MHC class I epitope during the acute and chronic phases of infection in a chimpanzee studied through seven years of follow-up. A C-terminal epitope of the NS3 protein, NS3_1629–1637_, restricted by the chimpanzee Patr-B1701 molecule, has previously been shown to serially acquire several distinct mutations in amino acid residues that impair MHC binding or TCR recognition [15],[35],[36]. The availability of longitudinal samples from this chimpanzee facilitated a careful examination of epitope evolution and an integrated assessment of the fitness of viral variants that arose in vivo, as well as the host immune response directed against these variants. Our results indicate that genomes encoding CTL escape mutations that emerge early in infection are not necessarily optimized for replication and are eventually replaced by variants that successfully balance escape from cellular immune pressure and replicative fitness in the chronic phase of infection. We predict that this could be an important factor influencing virus load in HCV-infected chimpanzees and humans, with as yet unknown consequences for liver disease progression.

## Materials and Methods

### Patr-B1701 plasmid, subgenomic replicon, and full-length chimeric genome construction

#### Patr-B1701 plasmid

The Patr-B1701 sequence was cloned into the pcDNA3.1Zeo(-) plasmid (Invitrogen, Carlsbad, CA). Briefly, the Patr-B1701 sequence was cloned from an EBV-transformed B cell line generated from chimpanzee CH503, linearized using SalI-EcoRI (1337 bp), and ligated to the multiple cloning site (MCS) of pcDNA3.1Zeo(-) cut with XhoI-EcoRI. Ligations were transformed into DH5α cells (Invitrogen, Carlsbad, CA), plated, and colonies picked and sequenced using the T7 forward and BGH reverse priming sequences (Macrogen, Korea).

#### Subgenomic replicons

Subgenomic replicons have been previously described [29],[30]. The original BB7 replicon backbone containing a neomycin cassette under the control of the 5′ HCV internal ribosomal entry site (IRES) and the NS3-NS5B genes under the control of an EMCV IRES was modified using site-directed PCR mutagenesis and standard cloning procedures, as described below.

#### JFHxJ6 Cp7 NS3 mutants

Plasmids pJFH1 (JFH) and pGND (GND) have been previously described [33], and plasmid pJ6CF (J6) is an autologous genotype 2a full-length clone that has been shown to be infectious in chimpanzees [37]. Sequence homology for NS3 between the infecting HCV 1/910 strain and BB7 is 92.2% and between HCV 1/910 and JFH/J6 is 80%. Proper controls to assess the effects of these differences at the epitope level were engineered into the backbones of BB7 and Cp7, respectively. To create the full-length chimeric JFHxJ6 Cp7 (Cp7) construct, two PCR products were produced and fused to create junction points between Core-p7 of J6 and p7-NS5B of JFH, resulting in the JFHxJ6 Cp7 full-length clone (described in [34]). NS3_1629–1637_ mutations were introduced in the Cp7 clone as follows, using standard cloning procedures. A single forward primer “039NS3Epi Forward” (5′-GATTCCCCTATCCTGCATCAAG-3′) was used with four reverse primers “040NS3EpiGAVQNEITL” (5′-GTCAGCTTGCATGCATGTGGCGATGTACTTCGTCCCAGGGTGTGTGAGGGTAATCTCATTTTGTACAGCGCCCAAACGGTACAGGAGAGG-3′), “041NS3EpiGAVQNEITP” (5′-GTCAGCTTGCATGCATGTGGCGATGTACTTCGTCCCAGGGTGTGTAGGGGTAATCTCATTTTGTACAGCGCCCAAACGGTACAGGAGAGG-3′), “042NS3EpiGAVQNEITS” (5′-GTCAGCTTGCATGCATGTGGCGATGTACTTCGTCCCAGGGTGTGTGCTGGTAATCTCATTTTGTACAGCGCCCAAACGGTACAGGAGAGG-3′), and “043NS3EpiGAVQNETTL” (5′-GTCAGCTTGCATGCATGTGGCGATGTACTTCGTCCCAGGGTGTGTGAGGGTGGTCTCATTTTGTACAGCGCCCAAACGGTACAGGAGAGG-3′), to amplify mutated NS3_1629–1637_ epitopes from the Cp7 full-length genome. PCR fragments were gel purified, NsiI-SacI digested (819 bp fragment cut to a 737 bp fragment), and a three-piece ligation performed; NsiI-SacI epitope fragment+NsiI-AvrII Cp7+AvrII-SacI-BbvCI Cp7. All fragments generated by PCR were verified by sequencing (Macrogen, Korea).

### Peptides

Wild-type (GAVQNEITL) and mutant (GAVQNEITP, GAVQNEITS, GAVQNETTL) NS3_1629–1637_ peptides were synthesized by Genemed Biosynthesis (San Francisco, CA) and purified by high-performance liquid chromatography (HPLC). All peptides were stored at a concentration of 1 mg/ml at –20°C.

### Cell lines and cell culture

Huh-7.5 cells were maintained in Dulbecco's modified Eagle's medium supplemented with 10% fetal bovine serum (FBS, Hyclone, Logan, UT) at 37°C in 5% CO_2_. The Huh-7.5/B1701 cell line was generated as follows. Huh-7.5 cells were trypsinized, washed with DMEM-10 media, and 4×10^6^ cells electroporated with 2.5 µg pcDNA3.1Zeo(-)B1701 plasmid using the AMAXA T-028 program (AMAXA, Gaithersburg, MD). Cells were resuspended in DMEM-10, plated in a p100 petri dish, and allowed to rest for 24 h before addition of 300 µg/ml zeocin (Invitrogen, Carlsbad, CA). Cell foci surviving selection were trypsinized, transferred to a 24-well plate, and allowed to grow under selection up to a p150 petri dish. Huh-7.5/B1701 cells were stained with 20 µl of anti-human pan-HLA-A, B, C FITC-conjugated antibody (BD Biosciences, San Jose, CA) in FACS buffer (0.5% (w/v) bovine serum albumin+1% (v/v) of 10% sodium azide in PBS) in parallel with untransfected Huh-7.5 cells, and visualized on a Becton Dickinson FACScalibur flow cytometer. Huh-7.5 and Huh-7.5/B1701 were transfected with replicons harboring wild-type or mutated NS3_1629–1637_ epitopes as previously described [29],[30]. The NS3_1629–1637_-specific CD8+ T cell clone has been previously described [15], and was stimulated using αCD3 mAb (Immunotech, Beckman Coulter, Fullerton, CA) in a ratio of 1×10^6^ CD8+ clone to 2×10^6^ irradiated peripheral blood mononuclear feeder cells (PBMC) and maintained in RPMI media supplemented with 10% FBS, Gentamicin (Gibco, Invitrogen, Carlsbad, CA), Penicillin/Streptomycin (Lonza, Walkersville, MD), T-stim culture supplement (human-no PHA, BD Biosciences, San Jose, CA) and human recombinant IL-2 (rIL-2, Roche, Indianapolis, IN). Autologous chimpanzee B cells were EBV-transformed following established protocols using whole blood and conditioned medium from the marmoset cell line B95-8 [38].

### Western blots for intracellular HCV protein

Huh-7.5 cells and Huh-7.5/B1701 cells with or without subgenomic replicons were lysed directly on 6-well plates using 150 µl lysis buffer (100 mM Tris pH 6.8, 20 mM dithiothreitol, 4% (w/v) sodium dodecyl sulfate, 20% (v/v) glycerol, 0.2% (w/v) bromophenol blue) and passed through a 27^1/2^ gauge needle 3–5 times before being stored at –80°C. Lysates were denatured at 92°C for 10 min, run on 5% stacking/8% resolving SDS-polyacrylamide gels, and transferred to Immobilon-P membranes (Millipore Corporation, Bedford, MA). Membranes were blocked with TBS-T (20 mM Tris pH 7.4, 150 mM NaCl, 0.1% (v/v) Tween-20 (polyoxyethylene sorbitan monolaurate) plus 5% (w/v) non-fat dry milk, and probed with antibodies against NS3 and NS5 (Virostat, Portland, ME), or β-actin in the same buffer overnight at 4°C. Membranes were washed 5 times with TBS-T, probed with HRP-conjugated secondary antibodies for 1 h at room temperature, washed 5 times, and detected using ECL Western detection reagents (Amersham Biosciences, Piscataway, NJ).

### Quantification of HCV RNA by real time qRT-PCR

Total RNA from 1×10^6^ infected Huh-7.5 cells was isolated using an RNeasy Mini Kit (QIAGEN, Valencia, CA). 80 ng of total cellular RNA was used to perform Real-Time Quantitative Reverse Transcription PCR using Taqman® One Step RT-PCR Master Mix Reagents (Applied Biosystems, New Jersey, USA), primers specific for the HCV 5′ NTR (forward, 10 µM: 5′-CTTCACGCAGAAAGCGCCTA-3′ and reverse, 10 µM: 5′-CAAGCGCCCTATCAGGCAGT-3′), and a probe (10 µM: 6-FAM-TATGAGTGTCGTACAGCCTC-MGB NFQ). Thermal cycling conditions were designed as follows: 48°C for 30 min, 95°C for 10 min, and 40 cycles of 15 s at 95°C, followed by 1 min at 60°C. All amplification reactions were carried out in duplicate. A standard curve was similarly generated using 10-fold dilutions of pJFH1 RNA transcripts generated by in vitro transcription, DNAse treatment, purification by RNeasy Mini Kit and quantification by spectrophotometry.

### ^51^Cr-release assay

To determine lysis capability of the NS3_1629–1637_-specific CD8+ T cell clone, Huh-7.5/B1701 cells with or without subgenomic replicons and EBV-transformed autologous B cells were spun at 1500 rpm for 5 min in a Beckman Coulter Allegra X-15R centrifuge, media aspirated, and tubes vortexed to resuspend the pellet. Cells were pulsed with ^51^Cr per standard protocol (NEN Radiochemicals, Perkin Elmer, Waltham, MA) for 1 h, and cells not harboring subgenomic replicons were simultaneously pulsed with 1 µg/ml wild-type NS3_1629–1637_ peptide resuspended in a total volume of 100 µl RPMI-10. Pulsed cells were washed 5 times to eliminate residual radiation and exogenous peptide, and mixed at different effector (NS3_1629–1637_-specific CD8+ T cell clone) to target ratios in 200 µl RPMI-10 in a 96-well round-bottom plate. Lysis was allowed to occur for 4 h at 37°C in 5% CO_2_ before transferring 100 µl of supernatant to a flat-bottom 96-well plate. Supernatants were frozen at –80°C for at least 1 h to eliminate cellular carryover before being counted using a 1450 Microbeta Wallac Trilux liquid scintillation counter (Perkin Elmer, Waltham, MA).

### Transfection and infection of human hepatoma cell lines

To transfect viral RNA, 20 µg of full-length JFHxJ6 Cp7 genomes with or without NS3_1629–1637_ mutations were linearized by 4 h digestion with XbaI and subsequently blunt end-digested with Mung Bean Nuclease (New England Biolabs, Ipswich, MA). Linearized DNA was extracted twice with 25∶24∶1 phenol∶choroform∶isoamyl alcohol pH5.2±0.2 and once with chloroform, quantified by spectrophotometry, and 2 µg of purified product was RNA transcribed using a MEGAscript T7 High Yield Transcription Kit (Ambion, Austin, TX). RNA was purified again using phenol∶chloroform∶isoamyl alcohol followed by chloroform, and integrity was checked on an agarose gel. After RNA quantification by spectrophotometry, Huh-7.5 or Huh-7.5/B1701 cells were trypsinized for exactly 3 min, washed twice with ice cold PBS, and resuspended at 2×10^7^ cells/ml. 10 µg of purified RNA was electroporated into 8×10^6^ cells with 5 pulses of 99 µs at 820 V over 1.1 s in an ECM 830 electroporator using a 2 mm-gap electroporation cuvette (BTX Genomics, Harvard Apparatus, Holliston, MA). Cells were resuspended in DMEM-10 and plated in 6-well plates. To infect Huh-7.5 or Huh-7.5/B1701 cells using whole virus, cells were plated at 10–20% confluency in six-well plates. Media was aspirated, and viral supernatants harvested from transfected cells were added to the plates in a volume of at least 200 µl. Plates were placed on a rocker at 37°C and 5% CO_2_ for 4 h before readdition of media.

### Huh-7.5/B1701 cells as APCs and intracellular IFNγ release assay

Huh-7.5 and Huh-7.5/B1701 that had not been electroporated (either with subgenomic replicons or full-length viral RNA) or infected with whole virus were pulsed for 1 h with wild-type or mutated NS3_1629–1637_ peptides at decreasing concentrations as in the ^51^Cr-release experiments. Cells harboring sugenomic replicons were harvested and used directly. Cells that had been transfected with full-length viral RNA were harvested 4 d post-transfection, and cells that had been infected with whole virus were harvested 5 d post-infection. Cocultures were established in a 24-well plate using a 1∶1 ratio of NS3_1629–1637_-specific CD8+ T cells to APCs, and allowed to incubate overnight at 37°C in 5% CO_2_ in the presence of GolgiStop (BD Pharmingen, San Jose, CA) at a concentration of 1 µl/ml. After incubation, cells were harvested, washed once in FACS buffer, and permeabilized using BD FACS Permeabilizing Solution 2 (BD Biosciences, San Jose, CA). Cells were stained with mouse anti-human monoclonal antibodies to CD3 (APC-conjugated, BD Pharmingen, San Jose, CA), CD8 (PerCP-conjugated, BD Biosciences, San Jose, CA), and IFNγ (FITC-conjugated, BD Pharmingen, San Jose, CA), and visualized on a Becton Dickinson FACScalibur flow cytometer. Data were analyzed using FlowJo software (Tree Star, Inc).

### Viral titration and immunohistochemical staining

96-well plates were coated with collagen for 1 h and allowed to dry before plating 6×10^3^ naïve Huh-7.5 cells/well. Viral supernatants from transfected or infected Huh-7.5 or Huh-7.5/B1701 cells were collected, passaged through a 0.22 µm filter, and used to inoculate cells at 10-fold dilutions. At 3 d post-infection, cells were immunostained for NS5A as previously described [32]. Briefly, the inoculum was removed, and cells were washed twice with PBS before fixation with methanol at –20°C. Cells were then washed twice with PBS, once with PBS+0.1% (v/v) Tween-20 (PBS-T) (normal wash), and blocked for 30 min at room temperature with PBS-T+1% (w/v) BSA+0.2% non-fat dry milk, followed by an endogenous peroxidase blocking step (3% H_2_O_2_ (v/v) in PBS) for 5 min at room temperature. Cells were washed normally and stained overnight at 4°C with an anti-NS5A antibody (9E10). Cells were washed normally, and incubated for 30 min at room temperature with a 1∶3 dilution of ImmPRESS goat anti-mouse HRP-conjugated antibody (Vector Laboratories, Burlingame, CA). Cells were washed normally once more before being developed using DAB substrate (Vector Laboratories, Burlingame, CA). Titers were determined by calculating the tissue culture infection dose at which 50% of wells were positive for NS5A antigen [39].

### Sequencing of viral clones

Huh-7.5/B1701 cells were infected using viral supernatants from day 4 transfected cells. Post-infection, media was aspirated, cells were washed twice with PBS and lysed using Buffer RLT (QIAGEN, Valencia, CA)+1% 2-mercaptoethanol (Fisher Scientific, Pittsburgh, PA). Lysates were placed directly onto QIAshredder columns, and total RNA isolated and purified using an RNeasy kit (QIAGEN, Valencia, CA). RNA integrity was quantified using a spectrophotometer, checked on an agarose gel, and 2 µg used in a first-strand cDNA synthesis reaction as follows. Briefly, RNA was incubated with random hexamer primers and 10mM dNTPs at 65°C for 5 min, then placed on ice. First-strand reverse transcriptase buffer, 0.1mM DTT and RNase H (Invitrogen, Carlsbad, CA) were added to each reaction and allowed to incubate at room temperature for 2 min before the addition of superscript II reverse transcriptase (Invitrogen, Carlsbad, CA). The reaction was allowed to proceed at 42°C for 2 h, and first-strand cDNA was used directly in an NS3_1629–1637_ epitope-specific PCR reaction using primers “211NS3EpiSeqInF” (5′-TCGCGTACCTAGTAGCCTACCAAGC-3′) and “212NS3EpiSeqInR” (5′-GCTGGTTGACGTGCAAGCGGCCGA-3′) to generate a 323bp fragment containing the epitope. PCR products were cleaned using a PCR cleanup kit (QIAGEN, Valencia, CA), cloned into Top10 chemically competent cells using a TOPO TA kit (Invitrogen, Carlsbad, CA), and individual clones were sent for sequencing (Macrogen, Rockville, MD).

### Polyclonal antigen-specific expansion of T cells and intracellular cytokine staining

CD8+ T cells were positively isolated from frozen PBMC using the Dynal CD8+ Positive Isolation Kit (Invitrogen Dynal AS, Oslo, Norway) according to manufacturer instructions. Approximately 360,000 CD8+ T cells were plated in one well of a 24-well plate in 1 ml complete medium (RPMI 1640 containing 10% AB human serum and 1% penicillin/streptomycin). To serve as APCs, 6 million irradiated autologous PBMC were pulsed for 2 h with 5 µg/ml of the GAVQNETTL peptide. After three washes, APCs were resuspended in 1 ml complete medium and mixed with the CD8+ T cells. Cells were incubated at 37°C in 7% CO_2_. Every 3 d, 1 ml of the culture medium was replaced with 1 ml of complete medium containing 50 U/ml rIL-2. On day 20, CD8+ T cells were plated in a 96-well plate in AIM-V medium (Aim-V (Invitrogen, Carlsbad, CA) supplemented with 2% AB human serum) and allowed to rest for 8 h. Irradiated EBV-transformed autologous B cells were pulsed for 2 h with 10 µg/ml of peptide for use as APCs. After three washes, APCs were resuspended in Aim-V medium and mixed with CD8+ T cells at a 1∶1 ratio with 1 µg/mL of anti-CD28 and anti-CD49d antibodies (BD Pharmingen, San Jose, CA). After 1 h, GolgiStop (BD Pharmingen, San Jose, CA) was added at a concentration of 1 µl/ml and cells were further incubated 16 h at 37°C in 7% CO_2_. After incubation, cells were harvested and washed once in FACS buffer. Cells were blocked with PBS-20% human serum and then stained with mouse monoclonal antibodies to CD8 (APC-conjugated, BD Pharmingen, San Jose, CA) and CD4 (Pacific Blue-conjugated, BioLegend). After two washes with FACS buffer, cells were stained with Live/Dead Fixable Blue Stain Kit (Invitrogen, Carlsbad, CA). Cells were washed twice with FACS buffer and permeabilized using BD Cytofix/Cytoperm solution (BD Biosciences, San Jose, CA). Cells were then stained with mouse monoclonal antibodies to CD3 (PerCP-conjugated, BD Pharmingen, San Jose, CA) and IFNγ (PE-conjugated, BD Pharmingen, San Jose, CA) and visualized on a Becton Dickinson LSR flow cytometer. Data were analyzed using FlowJo software (Tree Star, Inc).

## Results

### CTL escape mutations affect subgenomic transduction efficiency

**Figure 1:**
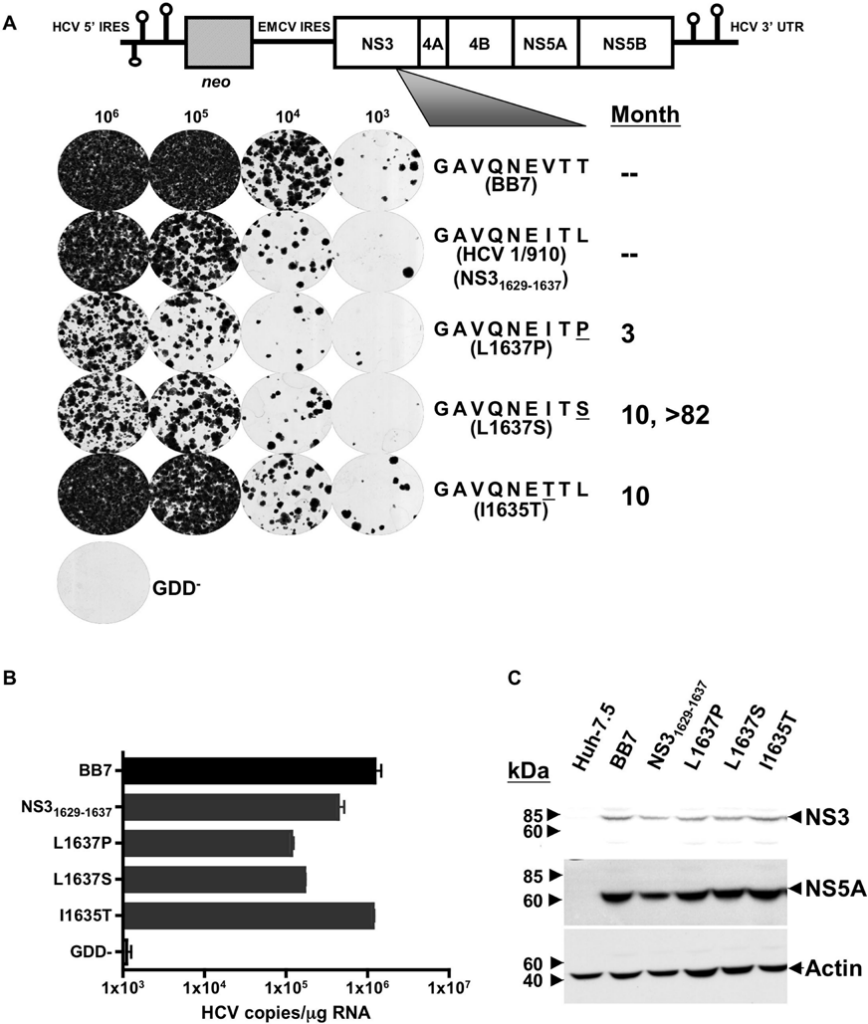
Construction of subgenomic replicons and HCV protein expression in transfected Huh-7.5 cells. (A) Schematic representation of subgenomic (SG) replicons and their replication efficiency. Huh-7.5 cells were transfected with the indicated constructs and plated at decreasing cell number concentrations under neomycin selection to determine transduction efficiency of each construct, with GDD^−^ serving as a negative control. NS3_1629–1637_ epitopes are listed by month(s) first detected in an in vivo chimpanzee CH503 infection model. (B) Replication of SG HCV RNA inside Huh-7.5 cells. Huh-7.5 cells were transfected with subgenomic replicons as in (A), and assayed for RNA replication 6 d post-transfection using a real time qRT-PCR Taqman assay as described in Materials and Methods. The minimum sensitivity of detection was 1,000 HCV copies/µg RNA). (C) Western blot of replicon-transfected Huh-7.5 cell lysates. The expression of HCV proteins NS3 and NS5A were detected post-transfection using anti-NS3/anti-NS5A monoclonal Abs. Lysates from Huh-7.5 cells transfected with replicons containing the BB7 epitope served as a positive control, untransfected Huh-7.5 cells were used as a negative control, and β-actin served as a positive control for input protein.

It is well established that the Patr-B1701 restricted NS3_1629–1637_ epitope is a dominant target of CD8+ T cells during HCV infection in chimpanzees [15],[36]. This epitope displayed a complex pattern of evolution throughout the acute and chronic phases of infection in the chimpanzee, and thus is valuable for the study of viral epitope escape and fitness costs associated with increased immune pressure. In chimpanzee CH503 infected with a known inoculum of HCV1/910, sequence analysis over seven years showed three distinct mutations at three separate timepoints tested in the NS3_1629–1637_ epitope [15]. The wild-type amino acid sequence of this epitope in the input HCV1/910 inoculum was GAVQNEITL (and from here on referred to as the “parent” epitope, NS3_1629–1637_), and three months post-infection a L1637P variant was found in the animal. Ten months post-infection, this variant had been replaced by two dominant species, I1635T and L1637S, at P7 and P9 respectively. Eventually, L1637S became fixed in this chimpanzee, and was the only variant recovered up to 82 months post-infection (Figure 1A). There is one nucleotide change from leucine to proline and one nucleotide change from proline to serine and hence two changes to occur to change leucine to serine; this perhaps provides mechanistic insight into the early appearance of L1637P and its later replacement by L1637S. We set out to test directly the fitness cost associated with the mutating NS3_1629–1637_ variants by modeling the in vivo infection using the HCV subgenomic replicon system [29],[30]. Using site-directed PCR mutagenesis, we engineered the mutants in the NS3_1629–1637_ region that had previously been observed in chimpanzees, starting with the original HCV1/910 parental epitope sequence. The mutated replicons on the BB7 backbone were transfected into Huh-7.5 cells, which were then plated under neomycin selection at decreasing cell numbers to determine transduction efficiency (Figure 1A). It is important to note that the original amino acid sequence of NS3_1629–1637_ epitope present in the BB7 subgenomic replicon is GAVQNEVTT, and was modified to insert the parental NS3_1629–1637_ epitope of HCV1/910. Substitution of the parental HCV1/910 NS3_1629–1637_ epitope resulted in a slight decrease of transduction efficiency in this replicon. The P9 mutations GAVQNEITP (L1637P) and GAVQNEITS (L1637S), which show a 400-fold decrease in B1701 binding capacity [15], showed increased susceptibility to neomycin, with L1637P growing the least efficiently. The P7 GAVQNETTL (I1635T) mutation was more resistant to selection than the HCV1/910 parental GAVQNEITL NS3_1629–1637_ sequence. These results suggest that mutations in this epitope at P9 severely hinder the replicative capacity of the virus, while an isoleucine to threonine substitution at P7 (I1635T) has no apparent effect. Additionally, a GND clone containing a mutation in the NS5B RNA-dependent RNA polymerase GDD motif thus ablating HCV RNA replication was used as a negative control. A real-time quantitative RT-PCR assay was used to quantify the level of HCV RNA replication 6 d post-transfection of Huh-7.5 cells using relevant transcribed RNAs. As shown in Figure 1B, levels of viral RNA replication correlated identically with the transduction efficiencies observed between the different constructs shown in Figure 1A. NS3 and NS5A protein expression for all constructs, including the original HCV replicon BB7 epitope GAVQNEVTT, was similar when assayed by Western blot (Figure 1C). Similar non-structural protein expression suggests that the NS3_1629–1637_ mutations may affect initiation of replication, with a steady-state accumulation of protein expression occurring once replication has been established. It has been shown that replicons under drug-induced selective pressure, such as G418, display high levels of HCV replication (1000–5000 positive strand RNA molecules), and therefore may show similar levels of protein expression [40]. However, under non-selective conditions, replicons having lower transduction efficiencies are lost more rapidly than those with high transduction efficiencies, and these differences may be reflected in viral protein levels. Although the P9 L1637S mutation resulted in partially reduced replicative capacity, the intermediate phenotype (between L1637P and I1635T) suggests that this mutation represents a balance between replicative fitness and CTL escape. This observation is interesting as the P9 L1637S mutation became a fixed quasispecies through 7 years of persistent replication in animal CH503. It is also important to note that cells harboring HCV replicons were additionally sequenced to ensure fidelity of previously inserted mutations. Sequencing of at least six clones from each subgenomic-bearing Huh-7.5/B1701 cell line (six, nine, ten, and six clones for NS3_1629–1637_, L1637P, L1637S, and I1635T, respectively) showed no variation from expected amino acid sequences. This sequencing was carried out on PCR fragments spanning the inserted NS3_1629–1637_ epitope from total cellular RNA.

### Wild-type but not mutant NS3_1629–1637_ epitope is presented to NS3_1629–1637_-specific CTL on class I MHC Patr-B1701

**Figure 2:**
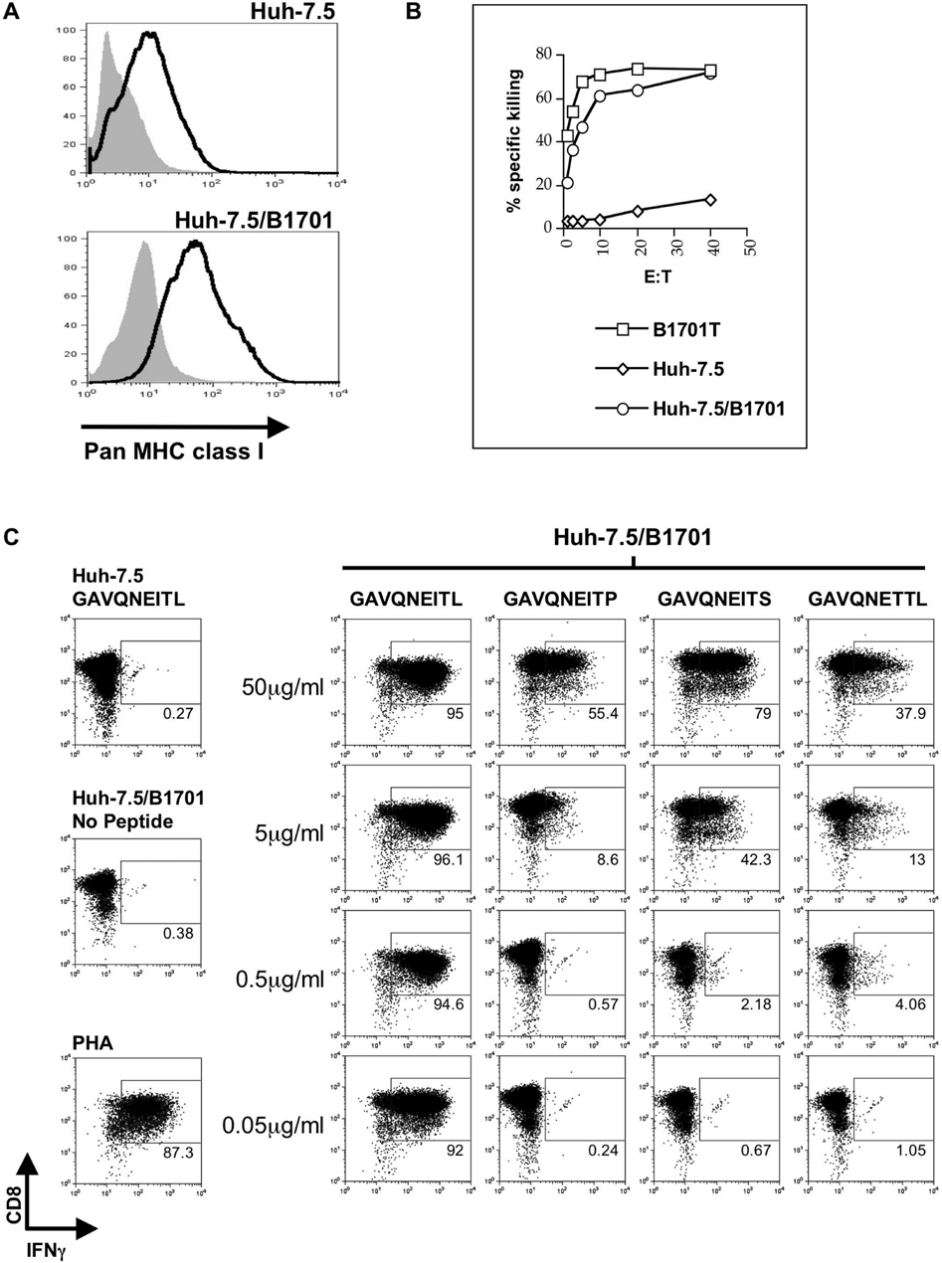
Expression and recognition of the chimpanzee Patr-B1701 molecule on the surface of Huh-7.5 cells. (A) Surface expression of MHC class I on Huh-7.5 cells transfected with a plasmid containing the Patr-B1701 molecule and zeocin selection marker. No difference in surface expression was observed when compared to untransfected Huh-7.5 cells. Isotype control is depicted in grey. (B) CTL lysis of transfected Huh-7.5 cells. Huh-7.5 cells expressing the Patr-B1701 (Huh-7.5/B1701) molecule were pulsed with wild-type peptide and incubated with increasing amounts of CTL clone 4A specific for the NS3_1629–1637_ wild-type epitope. Cells presenting this peptide on the Patr-B1701 molecule are lysed by CTLs as efficiently as EBV-transformed autologous B cells presenting peptide (B1701T). Untransfected Huh-7.5 cells served as a negative control. (C) CD8+ T cell clone IFNγ response to Huh-7.5/B1701 cells presenting exogenous peptide. Huh-7.5/B1701 cells were loaded with parent HCV1/910 NS3_1629–1637_ or mutant NS3_1629–1637_ peptide as in (B) and cocultured with a CD8+ T cell clone targeting the NS3_1629–1637_ epitope. Huh-7.5/B1701 cells presenting parent HCV1/910 NS3_1629–1637_ but not mutant peptide at concentrations of 0.5 µg/ml and lower could elicit an IFNγ response from the CD8+ T clone. Cocultures were stimulated with PHA as a positive control, and unpulsed Huh-7.5/B1701 cells or Huh-7.5 cells pulsed with parent HCV1/910 NS3_1629–1637_ peptide served as negative controls. Plots depicted are gated on CD3+ T cells.

We next sought to determine the ability of parental or mutated NS3_1629–1637_ epitope to be presented by a cell line expressing the appropriate chimpanzee MHC molecule. Patr-B1701 is a MHC class I molecule expressed in CH503, and previous data demonstrated that mutations at critical anchor residues in the NS3_1629–1637_ epitope (including P9) hindered peptide binding to Patr-B1701 [15]. We first examined pan-class I surface expression on Huh-7.5 cells, and compared those levels to Huh-7.5 cells that had been transfected with a plasmid containing Patr-B1701 under zeocin selection (Huh-7.5/B1701, Figure 2A). It is important to note that we are not able to specifically stain for the Patr-B1701 molecule since specific antibodies to this protein do not currently exist. However, overall class I expression was similar in both cell types when compared to the isotype control, which led us to test the ability of Patr-B1701 expression to mediate wild-type NS3_1629–1637_ epitope-directed killing by a B1701-restricted CTL clone, 4A, that had been previously isolated from CH503 11 weeks post-infection with the HCV-1/910 virus stock [15]. When EBV-transformed autologous B cells (B1701T) or Huh-7.5/B1701 cells were pulsed with exogenous wild-type NS3_1629–1637_ peptide (1 µg/ml) in a standard ^51^Cr-release assay, the NS3_1629–1637_-specific CTL clone was able to lyse both antigen presenting target cell populations with similar efficiency (Figure 2B). In contrast, peptide-pulsed Huh-7.5 cells lacking the Patr-B1701 molecule were not recognized by the CTL clone. In addition, we further determined the magnitude of the T cell interferon response to both the wild-type and mutated epitopes. To do so, we performed an intracellular IFNγ FACS analysis on the CTL clone cocultured with various APCs that had been pulsed with various peptide concentrations (Figure 2C). Huh-7.5/B1701 cells that had been pulsed for 1 h with wild-type NS3_1629–1637_ peptide elicited a robust IFNγ response from the CTL clone. This response was not elicited by the three mutant epitopes at concentrations up to 0.5 µg/ml. Responses to each epitope could be seen at very high concentrations indicating that the CD8+ T cell clone could be stimulated to produce IFNγ even with mutants that had previously been shown to have lowered MHC-binding capacity [15] if the mutant was present at high (but not biologically significant) concentrations. These data collectively show that Huh-7.5 cells stably transfected with the Patr-B1701 molecule efficiently present exogenous wild-type peptide in vitro, and that an antigen-specific T cell clone is able to respond by secreting IFNγ and exerting its cytotoxic effect. Conversely, this CTL clone is unable to efficiently respond when Huh-7.5/B1701 cells present mutated exogenous NS3_1629–1637_ peptides reflective of in vivo viral species, containing a threonine substitution at P7 or a proline or serine substitution at P9, at physiologically relevant concentrations.

### Huh-7.5/B1701 cells harboring subgenomic HCV replicons present the NS3_1629–1637_ epitope to NS3_1629–1637_-specific CTL

**Figure 3:**
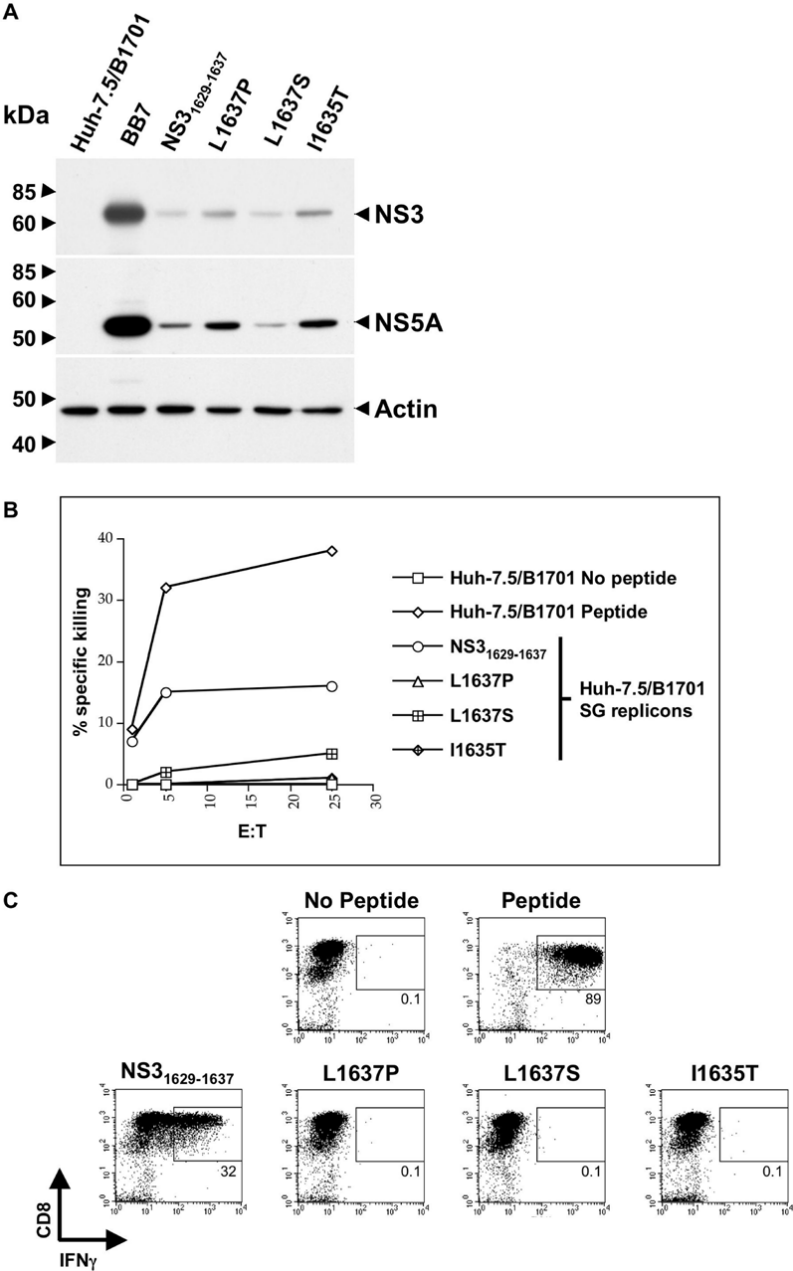
Mutations in the NS3_1629–1637_ epitope abrogate CTL recognition in the replicon system. (A) Western blots of replicon-transfected Patr-B1701-expressing Huh-7.5 cells. The expression of HCV proteins NS3 and NS5A were detected in Huh-7.5/B1701 cells harboring subgenomic replicons. Lysates from Huh-7.5/B1701 cells transfected with replicons containing the BB7 epitope served as a positive control, untransfected Huh-7.5/B1701 cells were used as a negative control, and β-actin served as a positive control for input protein. (B) CTLs are unable to lyse Huh-7.5/B1701 cells transfected with subgenomic mutant replicons with the same efficiency as the parent HCV1/910 NS3_1629–1637_ subgenomic replicon. Huh-7.5/B1701 cells with or without parent HCV1/910 NS3_1629–1637_ peptide were used as a positive and negative control, respectively. (C) CD8+ T cell IFNγ response to the mutated NS3_1629–1637_ epitope in the subgenomic system. CD8+ T cells specific for the wild-type NS3_1629–1637_ epitope were incubated with Huh-7.5/B1701 cells transfected with either the parent HCV1/910 NS3_1629–1637_ subgenomic replicon or the mutant subgenomic replicons, stained for CD8 and IFNγ, and analyzed by flow cytometry. CD8+ T cells secrete IFNγ in response to the parent HCV1/910 NS3_1629–1637_ subgenomic-transfected Huh-7.5/B1701 cells, but are unable to secrete IFNγ when incubated with Huh-7.5/B1701 cells harboring the subgenomic mutant replicons. Huh-7.5/B1701 cells were incubated with or without the parent HCV1/910 NS3_1629–1637_ peptide as a positive and negative control, respectively.

To determine whether Huh-7.5 cells expressing the Patr-B1701 expressed in CH503 could adequately process and present the NS3_1629–1637_ epitope during active viral replication for T cell recognition, Huh-7.5/B1701 cells were stably transfected with various HCV replicons containing the appropriate mutations under neomycin selection as before (see Figure 1A). Cells exhibiting zeocin resistance (confirming Patr-B1701 expression) and neomycin resistance (confirming presence of replicons) were used to determine protein expression as well as NS3_1629–1637_-specific CTL lysis and IFNγ production. Huh-7.5/B1701 cells harboring HCV replicons showed relatively similar levels of protein expression post-transfection and G418 selection (Figure 3A). Small differences in HCV protein expression were likely due to the stringency of dual selection (zeocin and G418) placed on these cells to maintain both the Patr-B1701 plasmid and the subgenomic construct. Protein expression was monitored to ensure that the replicons containing the parental HCV 1/910 NS3_1629–1637_ epitope and the mutants were able to produce, process and present similar levels of viral peptides to the CTL clone. Huh-7.5/B1701 cells harboring subgenomic HCV replicons were labeled with ^51^Cr, and cocultured with the NS3_1629–1637_-specific CTL clone to determine the ability of these cells to elicit epitope-directed lysis. Cells replicating the wild-type NS3_1629–1637_ replicon were lysed by the CTL clone, with ∼3-fold less efficiency than that seen using Huh-7.5/B1701 cells loaded with exogenous peptide (Figure 3B), reflective of lower levels of physiologic peptide generated in the replicon system. Cells harboring mutated NS3_1629–1637_ replicons elicited very low to undetectable levels of lysis, even at the highest effector to target ratios. Additionally, we assessed whether Huh-7.5/B1701 subgenomic cell lines replicating HCV RNA could elicit an IFNγ response from the NS3_1629–1637_-specific CTL clone. Similar to the pulsing experiment using low amounts of exogenous peptide shown in Figure 2C, only Huh-7.5/B1701 harboring the wild-type subgenomic replicon stimulated the CTL clone to produce IFNγ (Figure 3C).

### Infectious HCV harboring the wild-type NS3_1629–1637_ epitope but not escape mutants stimulate a functional T cell response

**Figure 4:**
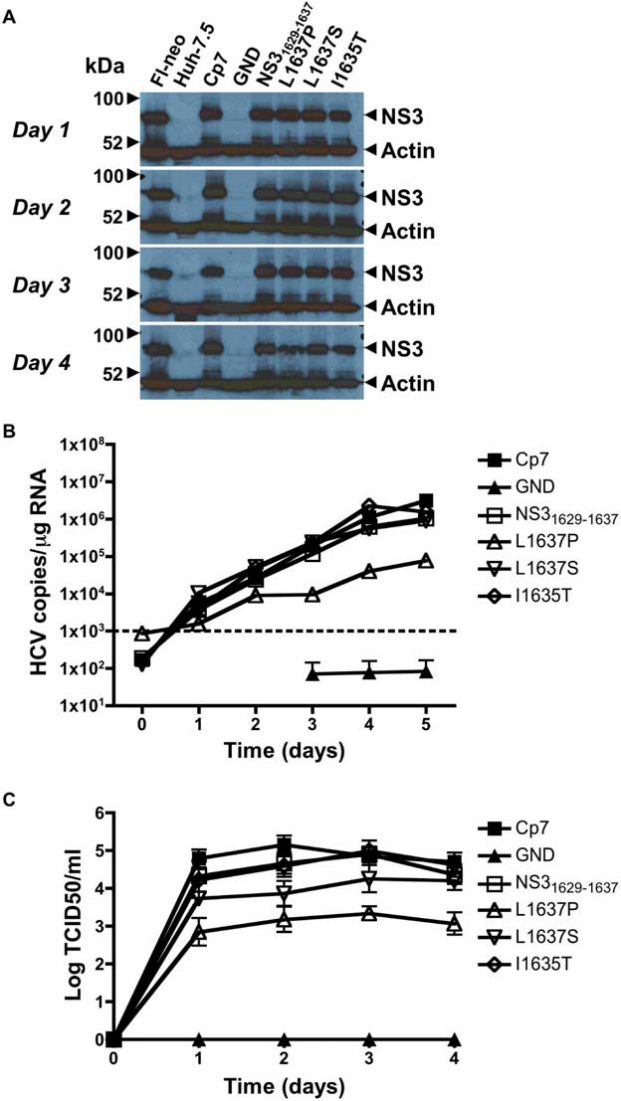
In vitro analysis of the mutated NS3_1629–1637_ epitope in a full-length viral genome system. (A) Short-term transfection Western blots. Expression of NS3 protein in transfected Huh-7.5 cells is similar among the mutant constructs. Fl-neo cell lysates harboring the full-length HCV genotype 1b replicon were used as a positive control, along with lysates from Huh-7.5 cells transfected with the Cp7 backbone construct. Untransfected Huh-7.5 cells and Huh-7.5 cells transfected with the replication-defective GND construct were used as negative controls. (B) Infection of naïve Huh-7.5 cells and quantitation of HCV RNA replication. Supernatants of transfected Huh-7.5 cells were harvested and normalized to infect naïve Huh-7.5 cells over a 5-d period with identical multiplicity of infection doses. Every 24 h, total RNA was harvested from cells and HCV RNA levels were measured using a qRT-PCR Taqman assay. Results are displayed as HCV copies/µg input RNA. The minimum sensitivity of detection (1,000 HCV copies/µg RNA) is displayed as a dashed line. (C) Short-term transfection viral titers. Supernatants of transfected Huh-7.5 cells were harvested up to 4 d post-transfection, and used to infect naïve Huh-7.5 cells. NS5A monoclonal antibody (9E10) was used in an immunohistochemical assay to determine TCID50/mL viral titers. Mean standard error bars from 4 separate experiments are displayed.

**Figure 5:**
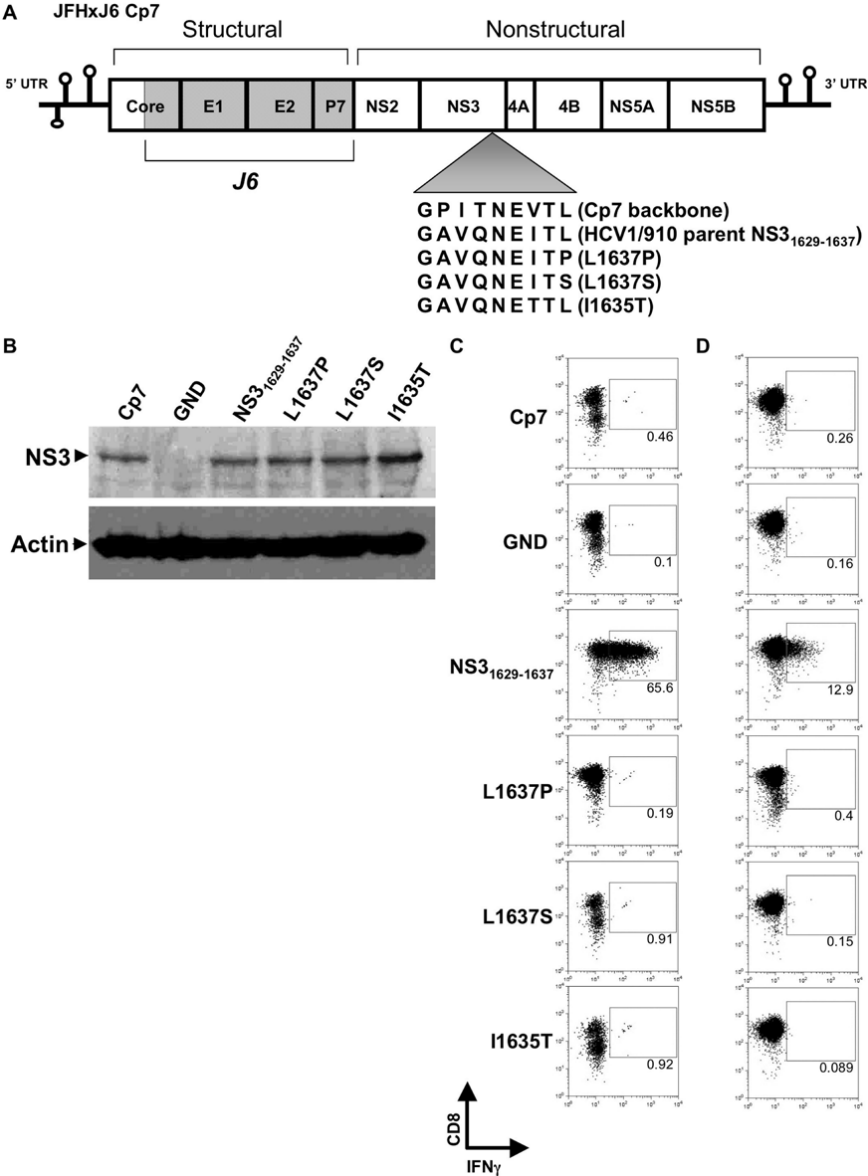
CD8+ T cell response to the mutated NS3_1629–1637_ epitope in an infectious system. (A) Schematic representation of the full-length HCV genome used to produce infectious virus in vitro. The core to p7 region of the JFH backbone was replaced by the corresponding region of the autologous genotype 2a J6 strain to create JFHxJ6 Cp7 (Cp7), and single-site PCR mutagenesis was used to alter the NS3_1629-1637_ epitope. (B) Western blot confirmation of NS3 protein expression 4 d post-transfection in the Huh-7.5/B1701 cell line. β-actin served as a control for input protein. (C) Transfection and recognition of Huh-7.5/B1701 cells by 4A CD8+ T cell clone. Huh-7.5/B1701 cells were transfected with parent HCV1/910 NS3_1629–1637_ or mutant full-length constructs, and cocultured (as in Figures 2C and 3C) with the NS3_1629–1637_ epitope-specific CD8+ T cell clone. The CD8+ clone was only able to secrete IFNγ in response to Huh-7.5/B1701 cells transfected with the parent HCV1/910 NS3_1629–1637_ construct. Huh-7.5/B1701 cells pulsed with parent HCV1/910 NS3_1629–1637_ peptide served as a positive control (not shown). (D) Infection of naïve Huh-7.5/B1701 cells with parent HCV1/910 NS3_1629–1637_ but not mutant full-length constructs elicits an IFNγ response. Supernatants were harvested from Huh-7.5/B1701 cells that had been transfected 4 d earlier and used to infect naïve Huh-7.5/B1701 cells. At 5 d post-infection, cells were harvested and cocultured with the CD8+ T cell clone to determine the level of IFNγ production produced by the 4A CD8+ T cell clone.

To test the replication fitness and infectivity costs associated with the mutations observed in the in vivo chimpanzee infection, we utilized the Huh-7.5/B1701 cell lines in both transfection and infection studies using full-length HCV constructs capable of producing infectious virus. The full-length genotype 2a JFH isolate has previously been shown to both replicate RNA and produce infectious virus in Huh-7 cells without acquiring adaptive mutations [33],[40]. To study robust replication and virion production in vitro, a recombinant clone was created by exchanging the core to p7 region of the genotype 2a JFH virus with the genotype 2a J6 virus, and the resulting JFHxJ6 Cp7 (Cp7) recombinant genome produces high titers of virus when used to transfect naïve Huh-7.5 cells (Figure 4C). As in the BB7 replicon system, the corresponding NS3_1629–1637_ epitope present in the JFH sequence of Cp7 was exchanged (using single-site PCR mutagenesis) with that of the parental HCV1/910, as well as the respective mutations seen in CH503 at 3, 10, and 82 months post-infection (Figure 5A). When RNA from parental NS3_1629–1637_ epitope and mutant viruses was transfected into Huh-7.5/B1701 cells, no major difference in protein expression was seen after 4 d as compared to the Cp7 backbone recombinant construct (Figure 5B). The GND transfected RNA expectedly did not produce any protein. These results are consistent with other experiments performed in both Huh-7.5/B1701 cells and Huh-7.5 cells in that these mutations do not affect overall expression of NS3 protein, but may affect initiation of replication (Figure 1C, 3A, 4A, 5B). To determine the efficiency of viral epitope processing and presentation by cells replicating full-length infectious HCV, the NS3_1629–1637_-specific CD8+ T cell clone was co-cultured with Huh-7.5/B1701 cells transfected with the Cp7 backbone, parental HCV1/910 NS3_1629–1637_, and individual mutant infectious clones. These T cells were then analyzed by flow cytometry to determine levels of IFNγ produced by the CD8+ T cell clone 4A. CD8+ T cells that had been stimulated by Huh-7.5/B1701 cells transfected with full-length infectious virus containing the wild-type NS3_1629–1637_ epitope had a robust intracellular IFNγ response, while those containing infectious virus with mutant epitopes were unable to elicit a T cell response (Figure 5C). To determine the level of NS3_1629–1637_ epitope presentation during actual viral infection, supernatants from transfected Huh-7.5/B1701 cells were harvested after 4 d, passed through a 0.22 µm filter, and used to infect naïve Huh-7.5/B1701 cells for 5 d. These cells were then cocultured with the NS3_1629–1637_-specific CD8+ T cell clone overnight, and examined via flow cytometry for IFNγ release. As previously shown with transfected cells, only Huh-7.5/B1701 cells infected with virus containing the wild-type NS3_1629–1637_ epitope were able to stimulate a response from the CD8+ T cell clone (Figure 5D).

### L1637P and L1637S mutations in the P9 anchor residue of NS3_1629–1637_ epitope impair viral fitness

Having previously established that mutations in the P7 and P9 residues of HCV NS3_1629–1637_ epitope ablate CD8+ T cell responses, we sought to determine the effect of each mutation on virion production. Cp7 backbone virus along with the parental HCV1/910 NS3_1629–1637_ and mutant viruses were transfected into Huh-7.5 cells, and protein expression assessed via Western blotting and virion production assessed using a TCID50/ml reinfection assay up to 4 d post-tranfection. There was little difference in protein expression between mutant, HCV1/910 NS3_1629–1637_, and Cp7 backbone infectious viruses (Figure 4A). At the level of RNA replication, as detected by a sensitive qRT-PCR Taqman assay, the viral variant L1637P replicated 1–1.5 logs less efficiently than parental virus (Figure 4B). Interestingly, L1637S replicated with similar efficiency to parental virus, indicating that this variant was competent in replication. However, several differences in virion production were observed. The leucine to proline switch in P9 of NS3_1629–1637_ epitope (L1637P) had a marked effect on the amount of virus secreted into the supernatant. This virus consistently produced 1.5–2 logs less virus than parental HCV1/910 NS3_1629–1637_ and Cp7 backbone virus (Figure 4C). The P7 isoleucine to threonine substitution (I1635T) produced similar levels of virus to the wild-type and Cp7 backbone, all secreting approximately 10^5^ TCID50/ml. However, the leucine to serine P9 substitution (L1637S) displayed increased virion production compared to the L1637P mutation, producing approximately 10^4^ TCID50/ml. The L1637S substitution of the parental HCV1/910 NS3_1629–1637_ epitope was found fixed in CH503 from month 10 post-infection up to 82 months post-infection (Figure 1A), and the recovered virion production phenotype seen in vitro suggests that L1637S is a more fit clone than L1637P, able to survive with intermediate fitness but efficient escape from immune elimination.

### L1637P reverts to the parental NS3_1629–1637_ epitope sequence while L1637S is stable in the absence of CD8+ T cell pressure

**Figure 6:**
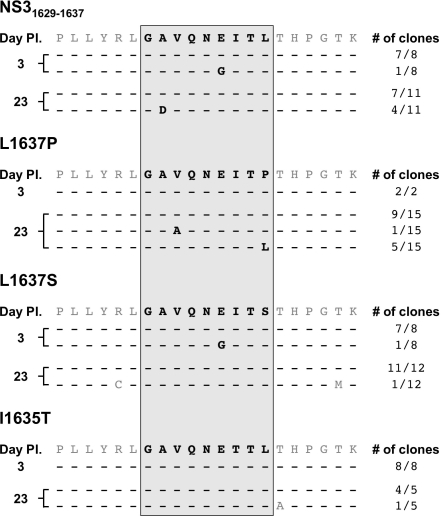
NS3_1629–1637_ epitope evolution during in vitro viral infection. At indicated times post-infection, cells were harvested and the nucleotide sequence of the viral NS3_1629–1637_ epitope was cloned and examined. Input sequence is listed above each timepoint, and “# of clones” denotes the number of individual colonies with the displayed sequence. Dashes represent no amino acid change from the listed input sequence.

Having established the relative fitness of each full-length viral clone, we wanted to determine what, if any, mutations arise in the NS3_1629–1637_ epitope during prolonged in vitro viral infection and replication. Huh-7.5/B1701 cells were infected with parental HCV1/910 NS3_1629–1637_ and mutant viruses, and cell lysates were used to obtain total cellular RNA up to 1 month post-infection. After first-strand cDNA synthesis and viral epitope-specific PCR, individual clones were sequenced. In the absence of CD8+ T cell selection pressure, we observed several amino acid mutations in the NS3_1629–1637_ epitope 3 d post-infection. For the parental NS3_1629–1637_ virus, one out of eight clones possessed a glutamic acid to glycine (E1634G) mutation at position 6 (Figure 6). This identical mutation was also found in the L1637S mutant virus 3 d post-infection. However, the E1634G mutation was absent in both the parental and L1637S viruses 23 d post-infection, indicating that this clone perhaps was not dominant or stable (Figure 6). Four out of eleven parental NS3_1629–1637_ clones harbored an A1630D mutation 23 d post-infection, which was unusual given the absence of CD8+ T cell pressure and the fitness of this virus in both the replicon (Figure 1A) and cell culture (Figure 4B,C) models. The I1635T mutant virus was stable over the infection course, exhibiting no mutations on both 3 and 23 d post-infection. Similarly, the L1637S mutant virus remained fixed 23 d post-infection, demonstrating the stability of this viral variant in our in vitro cell culture model. The stability of the L1637S mutant virus in vitro mirrors that which was observed in vivo with a serine at P9 stable 7 years post infection. Interestingly, the L1637P mutant virus was unchanged 3 d post-infection, but two variants were found 23 d post-infection (Figure 6). One variant, V1631A, was present at low frequency (1/15), while the other, P1637L, represented a significant fraction of the total population (5/15 clones). The P1637L mutation is particularly interesting because it represents reversion at position 9 in an unstable in vivo variant to the parental NS3_1629–1637_ epitope sequence in the absence of in vitro CD8+ T cell selection pressure. These results demonstrate that in vitro mutation of a CD8+ T cell epitope in hepatitis C virus can occur, that particular amino acid substitutions are not maintained over the course of in vitro infection, and that reversion of less fit viral variants to parental HCV sequence can occur when CD8+ T cell pressure is absent.

### An antigen-specific memory CD8+ T cell response prevents I1635T from becoming fixed in the viral population

**Figure 7:**
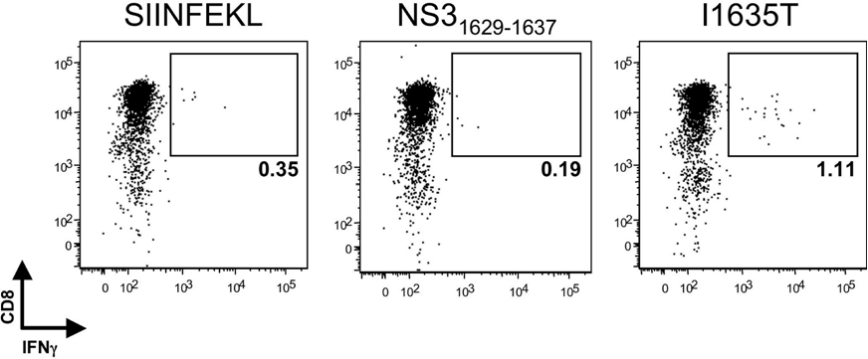
I1635T-specific CD8+ T cells are present in PBMCs from CH503 more than 7 y after infection. At 20 d post-expansion, 5×10^4^ CD8+ T cells were cultured with 5×10^4^ autologous EBV-transformed B cells pulsed with the indicated peptide, and the presence of peptide-specific IFNγ-secreting CD8+ T cells was assessed by intracellular cytokine staining. Cells are gated on live, CD3+ lymphocytes. The percent of CD8+ T cells that stained positively for intracellular IFNγ is displayed.

Variant I1635T was replication competent, escaped CTL recognition, and did not revert to the parental NS3_1629–1637_ sequence in the absence of selective pressure. Because the I1635T variant possesses a mutation in a TCR-contact residue (P7) and not an MHC-binding residue, we hypothesized that it may have stimulated a novel CD8+ T cell response in vivo, resulting in immune pressure preventing I1635T from becoming fixed in the viral population. To test this hypothesis, CD8+ T cells were isolated from frozen CH503 PBMC samples taken more than 7 years post-infection and stimulated with the I1635T peptide. Upon stimulation with autologous EBV-transformed B cells pulsed with I1635T peptide, CD8+ T cells secreted IFNγ (Figure 7). The I1635T antigen-specific CD8+ T cells did not respond to an irrelevant (SIINFEKL) control peptide, or the parental NS3_1629–1637_ peptide (Figure 7). These results support the hypothesis that although the I1635T variant replicated efficiently and escaped from NS3_1629–1637_-specific T cells, its persistence was hindered by a de novo T cell response.

## Discussion

Growing evidence has shown that CD8+ T cell epitope mutation and subsequent viral escape are associated with persistence of viruses like HIV, SIV, and HCV [17], [18], [26], [41]–[47]. Indeed, the rate of non-synonymous mutation leading to amino acid substitutions is statistically higher in MHC class I restricted epitopes than in non-restricted epitopes or flanking regions, indicating that they are subject to Darwinian selection pressure by CD8+ T cells [18], [26], [45], [48]–[53]. With the development of cell culture models that support HCV infection and replication, it is now possible to model how changes to the genome influence the rate of viral reproduction. In this study we have exploited these in vitro replication models to better understand how the potentially oppositional forces of immune evasion and efficient viral replication shaped evolution of a well-characterized dominant MHC class I epitope that displayed iterative adaptive mutations during establishment of HCV persistence in a chimpanzee. Mutational analyses in the currently available in vitro systems are limited by the necessity to study viral fitness and virion production in the context of genotype 1b and 2a backbones, respectively. However, even with the caveat that unpredictable coordinated effects of introducing epitopes from varying isolates may occur, the ability to study the consequences of single epitope serial sequence mutation on viral fitness and virion production is extremely valuable.

Generation of Huh-7.5 cells harboring subgenomic replicons allowed for primary analysis of viral RNA replication, and helped establish the initial fitness characteristics of each NS3 mutation that had been observed in vivo (Figure 1A and 1B). Even single amino acid changes in this epitope hindered transduction efficiency, with functional consequences of mutation on specific T cell responses. Interestingly, these changes did not seem to greatly affect protein expression, either in the replicon system or in the cell culture model (Figures 1C, 3A, 4A, and 5B). Similar non-structural protein expression implies that the NS3_1629–1637_ mutations may affect initiation of replication (fewer G418 resistant colonies per µg of RNA), but that once replication has been established similar levels of steady-state replication/protein accumulation would be observed. Similar protein expression levels should result in similar levels of viral peptide production.

The inability of P9 mutations to generate a T cell response could be overcome by high amounts of exogenous peptide but not by more physiologic concentrations generated by replicating subgenomic or full-length infectious viruses (Figures 3 and 5). By engineering the NS3_1629–1637_ epitope mutations into both a subgenomic replicon system and a recombinant full-length clone of Cp7 capable of robust virion production, a correlation between viral fitness and immune escape was established. CD8+ T cell recognition in transfected and infected Huh-7.5/B1701 cells occurred only with HCV1/910 parental NS3_1629–1637_ epitope (Figures 3B, 3C, 5C, and 5D), indicating that single amino acid changes in this epitope abrogate T cell recognition. Each of the single substitutions at P9 decreased virion production while the virus containing the observed mutation at P7 (I1635T) was unimpaired in virus production. Together with the observation that mutant L1637S was maintained over 7 years despite I1635T having better viral fitness, these results suggest a balance between efficient immune escape and virion production attained by L1637S mutant virus. That is, since the mutant epitope I1635T, with a threonine at T cell receptor contact residue P7, was detected at month 10 but not later in infection it is possible that its higher fitness and virion production allowed an additional T cell response to be generated against the new epitope. The I1635T mutation has been shown to bind well to Patr-B1701 [15], so that generation of novel CD8+ T cell clones targeting the I1635T epitope in vivo is plausible. In contrast, mutant L1637S, which abrogates MHC binding [15], may not select for a new T cell response to develop while still producing sufficient levels of virions. In fact in this study, using frozen PBMCs from CH503 from more than seven years after infection, we were able to isolate T cells specific for I1635T indicating that indeed, this otherwise “perfect” mutation was subject to new immune pressure in vivo (Figure 7). This de novo T cell response most likely prevented the I1635T variant from becoming stable in the population. Additionally, it is also possible that the P7 I1635T mutant isolated in vivo had fewer compensatory mutations in other highly targeted epitopes, allowing for recognition of the other epitopes by CD8+ T cells. Our data are consistent with a previous human study demonstrating that the variability of HCV sequences within immunological epitopes is limited by viral fitness [28], but extend these observations by assessing the long-term longitudinal evolutionary pattern of an immunologically and virologically important NS3 epitope.

It is noteworthy that the L1637P variant that appeared within 3 months of infection was least fit for replication in our cell culture models and was replaced in the plasma of the chimpanzee seven months later by two more fit variants. These results indicate that escaped viruses (like L1637P) may readily revert to a more fit sequence when transmitted from a recently infected donor to an HLA-mismatched recipient. In HIV-1, a CD8+ T cell-mediated escape mutation in the dominant HLA-B57 TW10 epitope (TSTLQEQIGW) within the capsid protein p24 has been shown to impair viral replication in vitro [23]. Reversion of this mutation following transmission to an HLA-mismatched host provided evidence for the impaired fitness cost that was incurred in vivo while escaping from CTL pressure [54]. Another study utilizing a clonal SIV virus (SIV_mac239_) harboring CTL escape mutations showed that escape can exact a severe replicative fitness cost, and that many of these variant sequences would be unlikely to propagate in HLA-diverse populations [51]. To date there are only two published examples of apparent reversion of escaped HCV epitopes in human subjects, and both involved viruses transmitted from donors during the acute phase of infection [18],[46]. Ray et al. followed a group of women infected with a common virus from a single acutely infected donor. When HCV genomes from the recipients were compared with a consensus HCV genome assembled from published sequences, mutations trending away from consensus were observed in HLA-restricted epitopes (representing possible emergence of recipient escape variants) and toward consensus in non-restricted epitopes (representing possible reversion of donor escape variants) [46]. That acute phase escape variants might revert to a more fit sequence is also supported by a second detailed study of CD8+ T cell immunity in a donor-recipient pair [18]. A CD8+ T cell escape mutation that arose during the acute phase of infection in the virus donor was quickly lost from the quasispecies upon transmission to an HLA class I disparate recipient [18]. Our results suggest that this reversion may be less common when mutations are optimized for immune escape and replication over long periods of chronic infection. We hypothesize that variants like L1637S that have been fine tuned by a process of iterative mutation during months of persistent replication might be considerably more stable upon transmission. We predict that these amino acid substitutions will not readily revert upon infection of a new host once escape from immunity has been carefully balanced against replicative fitness, particularly if HCV has a wide (though not limitless) tolerance for substitutions that alter replication. In vitro, we infected naïve Huh-7.5/B1701 cells with parental NS3_1629–1637_ and mutant viruses, and studied the epitope evolution of individual clones. Interestingly, we found mutations in numerous (5/15) clones of L1637P 23 d post-infection, with the P9 proline mutating back to the parental leucine (Figure 6). These results strengthen the hypothesis that the L1637P mutant virus has diminished replicative fitness in vitro as well as in vivo. Additionally, the absence of CD8+ T cell pressure in these experiments suggests that transiently less fit viruses may trend towards input parental sequence in HLA-diverse populations or upon transmission to HLA-mismatched hosts. Importantly, the L1637S and I1635T viruses were relatively stable, confirming replication and virion production data (Figures 1A, 1B, 4B, and 4C). The stability of L1637S suggests that this virus has indeed struck a balance between replicative fitness and immune pressure, and would not likely revert back to the parental sequence upon transmission to a new host. These data correlate with long term NS3_1629–1637_ epitope evolution in chimpanzees with L1637S stable over a 7-year period, and demonstrate that mutants arising in vivo can be recapitulated in vitro. We predict that the L1637S sequence represents a viral variant that has achieved balance between replicative capacity and immune evasion and would be stable upon transfer to naïve hosts regardless of whether they express MHC molecules required for presentation to CD8+ T cells.

The work reported here highlights the competing forces influencing the interplay between the virus and the immune system and the multiple varied effects of a single amino acid change on T cell function and virus production. These observations elucidate potential mechanisms by which viral persistence is established. Consequences of stable integration of escape mutations into viral genomes are not clear, but it is formally possible that epitopes presented by the most prevalent MHC class I molecules in human populations will eventually be lost or become less dominant, an outcome that could have implications for vaccine development. In light of the knowledge that HCV mutates nearly one nucleotide per replication cycle, this work provides sobering evidence that the anti-HCV CD8+ T cell response faces daunting challenges for efficient and lasting control of HCV.
